# Sero- and *apx*-typing of German *Actinobacillus pleuropneumoniae* field isolates from 2010 to 2019 reveals a predominance of serovar 2 with regular *apx*-profile

**DOI:** 10.1186/s13567-020-00890-x

**Published:** 2021-01-20

**Authors:** Lukas Schuwerk, Doris Hoeltig, Karl-Heinz Waldmann, Peter Valentin-Weigand, Judith Rohde

**Affiliations:** 1 Institute for Microbiology, Department of Infectious Diseases, University of Veterinary Medicine Hannover, Foundation, Hannover, Germany; 2 Institute for Pathology, University of Veterinary Medicine Hannover, Foundation, Hannover, Germany; 3 Clinic for Swine and Small Ruminants, Forensic Medicine and Ambulatory Service, University of Veterinary Medicine Hannover, Foundation, Hannover, Germany

**Keywords:** *Actinobacillus* *pleuropneumoniae*, Serotyping, *apx*, Virulence, Atypical, Biovar, Systemic

## Abstract

Serotyping is the most common method to characterize field isolates of *Actinobacillus (A.) pleuropneumoniae*, the etiological agent of porcine pleuropneumonia. Based on serology, many farms seem to be infected and antibodies against a wide variety of serovars are detectable, but, so far it is unknown to what degree respective serovars contribute to outbreaks of clinical manifest disease. In this study, 213 German *A.* *pleuropneumoniae* field isolates retrieved for diagnostic purposes from outbreaks of porcine pleuropneumonia between 2010 and 2019 were genetically serotyped and analyzed regarding their *apx*-toxin gene profile using molecular methods. Serotyping revealed a prominent role of serovar 2 in clinical cases (64% of all isolates) and an increase in the detection of this serovar since 2010 in German isolates. Serovar 9/11 followed as the second most frequent serovar with about 15% of the isolates. Furthermore, very recently described serovars 16 (n = 2) and 18 (n = 8) were detected. Most isolates (93.4%) showed *apx*-profiles typical for the respective serovar. However, this does not hold true for isolates of serovar 18, as 75% (n = 6) of all isolates of this serovar deviated uniformly from the “typical” *apx*-gene profile of the reference strain 7311555. Notably, isolates from systemic lesions such as joints or meninges did not harbor the complete *apxICABD* operon which is considered typical for highly virulent strains. Furthermore, the extremely low occurrence (n = 1) of NAD independent (biovar II) isolates in German *A.* *pleuropneumoniae* was evident in our collection of clinical isolates.

## Introduction

The etiological agent of porcine pleuropneumonia*, A.* *pleuropneumoniae,* is a bacterial pathogen belonging to the family of *Pasteurellaceae,* affecting pigs worldwide [1]. The bacterium is also contributing to porcine respiratory disease complex (PRDC), one of the most important economic challenges in pig farming [2]. Infections with *A.* *pleuropneumoniae* are well known to be associated with lesions of porcine pleuropneumonia (fibrino-hemorrhagic pleuropneumonia). Incidentally, systemic spreading of the pathogen in the host with associated lesions (e.g. arthritis, meningitis) is reported and thought to be due to hematogenic and/or lymphogenic dissemination [3–5]. *A.* *pleuropneumoniae* usually exhibits nicotinamide adenine dinucleotide (NAD)-dependent growth (biovar I), but NAD-independent strains of the bacterium have also been described (biovar II) [6].

The bacterium is characterized by the occurrence of numerous serovars, which can be distinguished by the expression of different capsular antigens and which are associated with different courses of disease [7]. Therefore, serovar-based pathogen typing is the most important means of diagnostic differentiation between isolates, and is essential for vaccination, as only limited cross-protection between different serovars occurs [8]. However, more and more serovars have been found over the years, resulting in the description of 18 different serovars so far [9, 10]. Due to problems of serotyping using serovar-specific antisera, such as limited reproducibility and cross-reactions, more reliable molecular genetic methods have been developed in recent years [11, 12]. However, molecular serotyping including all known serovars has not yet been applied to a collection of field isolates from Germany, one of the major global pig producing countries. The monitoring of serovar frequencies is essential for establishing effective control measures.

Among other virulence mechanisms, the RTX-toxins Apx I-III represent major virulence factors, which are substantially involved in development of disease. Apx I is strongly hemolytic and cytotoxic, while Apx II shows weak hemolytic and cytotoxic activity. In contrast, Apx III is considered to have no hemolytic, but strong cytotoxic effects on alveolar macrophages and neutrophils [13]. A fourth toxin (Apx IV) is found in all *A.* *pleuropneumoniae* isolates and thus represents a species-specific marker [14]. Differences in the virulence potential of different serovars can be partly explained by the (alleged) serovar-specific production of Apx toxins [15, 16]. Therefore, the virulence of *A.* *pleuropneumoniae* is considered to be closely associated with the serovar [13]. The description of the serovar-specific production of Apx toxins dates back to the 1990s. Since then, however, reports of atypical isolates of the bacterium have been published repeatedly [17–21] but the relevance of these findings is unclear.

Given the current knowledge gaps described above, this study was designed to characterize a comprehensive collection of *A.* *pleuropneumoniae* field isolates over the last decade with respect to serovar, biovar and *apx*-gene profile. These investigations shed light on the by that time unclear attributes of *A. pleuropneumoniae* field isolates and allow a more detailed monitoring and more efficient control of this important pig pathogen.

## Materials and methods

### Isolates

The isolates of *A.* *pleuropneumoniae* investigated in this study were obtained from diagnostic submissions to the Institute for Microbiology, University of Veterinary Medicine Hanover, Germany, originating mainly from northern, western and southern parts of Germany (see Additional file 1). A total of 213 isolates dating from the years 2010–2019 were examined. Over the years some farms were represented repeatedly. Isolates from these repeatedly sampled farms were included in the study if they belonged to different serovars or *apx*-genotypes. If isolates belonged to the same serovars and *apx*-genotype, they were only included if the date of isolation was more than 6 months apart which is consistent with the turnaround time of pigs on farms in Germany. Five older isolates (1999–2009) represented an additional collection of *A.* *pleuropneumoniae*. The strains used as controls for the molecular tests are shown in Table 1. For each molecular method, representatives of close relatives of *A.* *pleuropneumoniae* were also used to show the species-specificity of the applied methods. All isolates of *A.* *pleuropneumoniae* were grown on chocolate agar with 0.07% NAD (Columbia agar base, Thermofisher, Germany, with sheep blood added at 80 °C and NAD, VWR, Germany, added at 50 °C) and identified by detection of *apxIVA* [14]. Isolates of the years 2010–2019 were divided into three cohorts encompassing one-third (n = 71) of them each (cohort A dating from 2010–March/2015; cohort B dating from April/2015–February/2018 and cohort C dating from February/2018–2019).

**Table 1:** Control strains used in this study

Species	Strain	Serovar	*apx* profile
*A.* *pleuropneumoniae*	APP/04	1	*ICA, IBD, IICA*
*A.* *pleuropneumoniae*	ATCC27089	2	*IBD, IICA, IIICA, IIIBD*
*A.* *pleuropneumoniae*	ATCC27090	3	*IICA, IIIBD, IIICA*
*A.* *pleuropneumoniae*	M62	4	*IBD, IICA, IIICA, IIIBD*
*A.* *pleuropneumoniae*	K17	5a	*ICA, IBD, IICA*
*A.* *pleuropneumoniae*	L20	5b	*ICA, IBD, IICA*
*A.* *pleuropneumoniae*	ATCC33590	6	*IBD, IICA, IIICA, IIIBD*
*A.* *pleuropneumoniae*	WF83	7	*IBD, IICA*
*A.* *pleuropneumoniae*	405 Ireland	8	*IBD, IICA, IIICA, IIIBD*
*A.* *pleuropneumoniae*	CVJ 13261	9	*ICA, IBD, IICA*
*A.* *pleuropneumoniae*	D13039	10	*ICA, IBD*
*A.* *pleuropneumoniae*	56153	11	*ICA, IBD, IICA*
*A.* *pleuropneumoniae*	8329	12	*IBD, IICA*
*A.* *pleuropneumoniae*	N273	13	*IBD, IICA*
*A.* *pleuropneumoniae*	3906	14	*ICA, IBD*
*A.* *pleuropneumoniae*	HS 143	15	*IBD, IICA, IIIBD, IIICA*
*A.* *pleuropneumoniae*	A-85/14	16	*ICA, IBD, IICA*
*A.* *pleuropneumoniae*	16287-1	17	*IBD, IICA*
*A.* *pleuropneumoniae*	7311555	18	*IBD, IICA*
*A. minor*	8590/4/08		
*A. porcinus*	6416/3/04		
*A. indolicus*	3753/11/08		
*A. porcitonsillarum*	4931/04		

Serovar 13 was used as positive control for *nadV.*

### Serotyping

Molecular serotyping was carried out according to the method described by Bossé et al. [11]. For better differentiation of the PCR products in the agarose gel, this procedure was applied in three sequential steps. Step I included testing for serovars 1, 2, 10, 13 & 14; step II for serovars 3, 5, 7, 12, 15, 16 & *nadV* and step III for serovars 4, 6, 8, 9/11, 17 & 18. The respective reference strains of *A.* *pleuropneumoniae* were used as positive controls as well as the reference strain for serovar 13 as control for the detection of *nadV*. PCR were carried out using Qiagen HotStart Taq DNA Polymerase (Qiagen, Hilden, Germany) and the standard reaction set up for a 25 µl reaction volume including a final concentration of 1.5 mM MgCl_2_ and 200 µM of each dNTP (Carl Roth, Karlsruhe, Germany). Primers (Biomers, Ulm, Germany) were used at a final concentration of 0.3 µM each. Cycling conditions were 15 min at 95 °C for activation of Taq, 30 s at 95 °C for denaturation, 90 s at 56 °C for annealing and 2 min at 72 °C for elongation. A final extension step at 72 °C for 10 min was programmed and a total of 30 cycles were run on a Bio-Rad T100 cycler (Bio-Rad, Munich, Germany). 16 µl PCR product was run in a 2% agarose gel for 90 min for pattern analysis.

### *apx*-typing

The virulence typing of *A.* *pleuropneumoniae* was performed by genetic detection of the *apx* genes as described by Sthitmatee et al. [22]. Cycling conditions were 15 min at 95 °C for activation of Taq, 30 s at 94 °C for denaturation, 30 s at 59 °C for annealing and 2 min at 72 °C for elongation. Final primer concentrations in the PCR were 0.75 µM for *apxIBD* & *apxIICA* and 0.25 µM for *apxICA*, *apxIIICA* & *apxIIIBD*. All other reaction components and cycling parameters were the same as above. In contrast to serotyping, only 8 µl of the PCR product was used for a two-hour gel electrophoresis in a 1.5% agarose gel. As positive control, a common template (mixing ratio 1:1) of the reference strain for serovar 1 (*apxICA*, *apxIBD*, *apxIICA*) and for serovar 2 (*apxIBD*, *apxIICA*, *apxIIICA*, *apxIIIBD*) was used to cover all detectable virulence genes.

### Biovar examinations

Pathogen identification included testing of growth of isolates on Columbia sheep blood agar in co-cultivation with a *Staphylococcus hyicus* strain, which served as a “nurse” by providing NAD. Isolates of biovar I only grow in close proximity to the *Staphylococcus* strain, while those of biovar II can be cultivated independently of the “nurse”. Isolates suspect of biovar II were subcultured on brain heart infusion agar (BHI, Thermofisher, Germany) to demonstrate independency of any NAD residuals or precursor molecules in blood containing media. In addition, a molecular examination based on the detection of the *nadV* gene was also used for biovar identification. Primers for the detection of *nadV* were described by Bossé et al. and integrated in the second panel of the serotyping procedure (0.3 µM final concentration) [11].

## Results

### Serotyping

**Figure 1:**
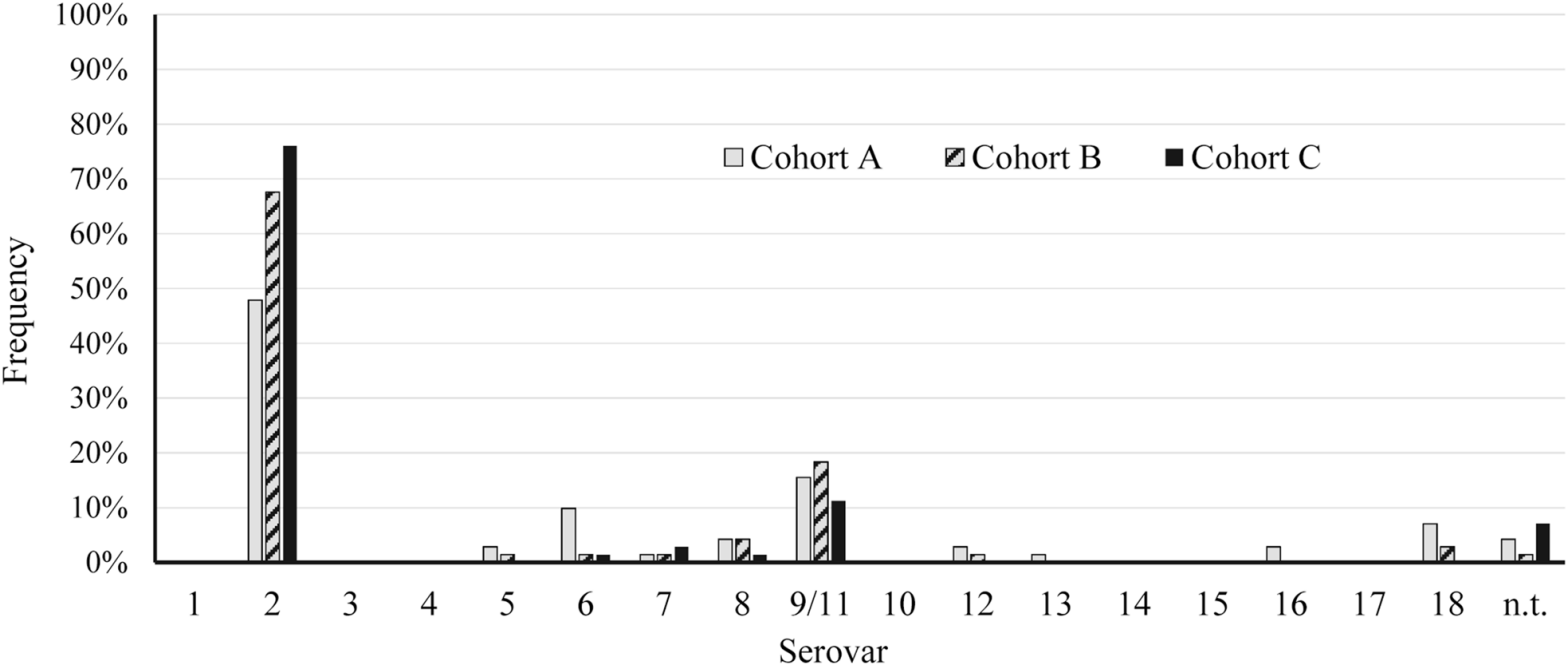
**Frequency of the serovars of** ***A.*** ***pleuropneumoniae*** **isolated between 2010 and 2019 (n = 213).** Cohort A: 2010–March 2015, Cohort B: April 2015–February 2018, Cohort C: February 2018–2019 encompassing 71 isolates each. n.t. = non-typable.

When looking at the results of serotyping, close to two-thirds (64%, n = 136) of the collection belonged to serovar 2. The second most frequent serovar was serovar 9/11 with about 15% of the isolates (n = 32), whereas other serovars were detected only occasionally. Serovars 5, 6, 7, 8, 12 and 13 together contained about 12% of the isolates, whereas 4% of the collection (n = 9) were non-typable (see Figure 1). Most interesting was the detection of serovars 16 (n = 2) and 18 (n = 7), with one of the additional isolates also belonging to serovar 18 originating from 2009.

Concerning the occurrence of serovars over the decade studied, individual changes in serovar composition in the isolate cohorts were observed (Figure 1). In cohort A (2010–March/2015) 48% of the isolates were serovar 2, which in cohort B (April 2015-February/2018,) accounted for 68% and in cohort C (February/2018–2019) for 76% of the isolates. Based on our grouping, this means a notably higher proportion of serovar 2 in cohort C compared to cohort A.

### *apx*-typing

In general, the toxin gene profiles corresponded well to the respective serovar, as 93.4% of the isolates (n = 199) showed a toxin gene profile typical for the respective serovar. In 2 of 7 isolates of serovar 18 an identical *apx* profile could be detected compared to the reference strain 7311555 (*apxIBD* and *apxIICA* only), thus the profile of these two isolates was interpreted as typical. The two isolates of serovar 16 also showed an expected *apx* profile with *apxICABD* and *apxIICA*. In addition, all non-typable isolates of the collection (n = 9) revealed *apx* gene profiles described for certain serovars: *apxICABD* and *apxIICA* (typical for serovars 1, 5, 9, 11 and 16) could be detected in six non-typable isolates and *apxIBD* and *apxIICA* (typical for serovars 7, 12, 13, 17 and 18) in the remaining three representatives.

**Figure 2:**
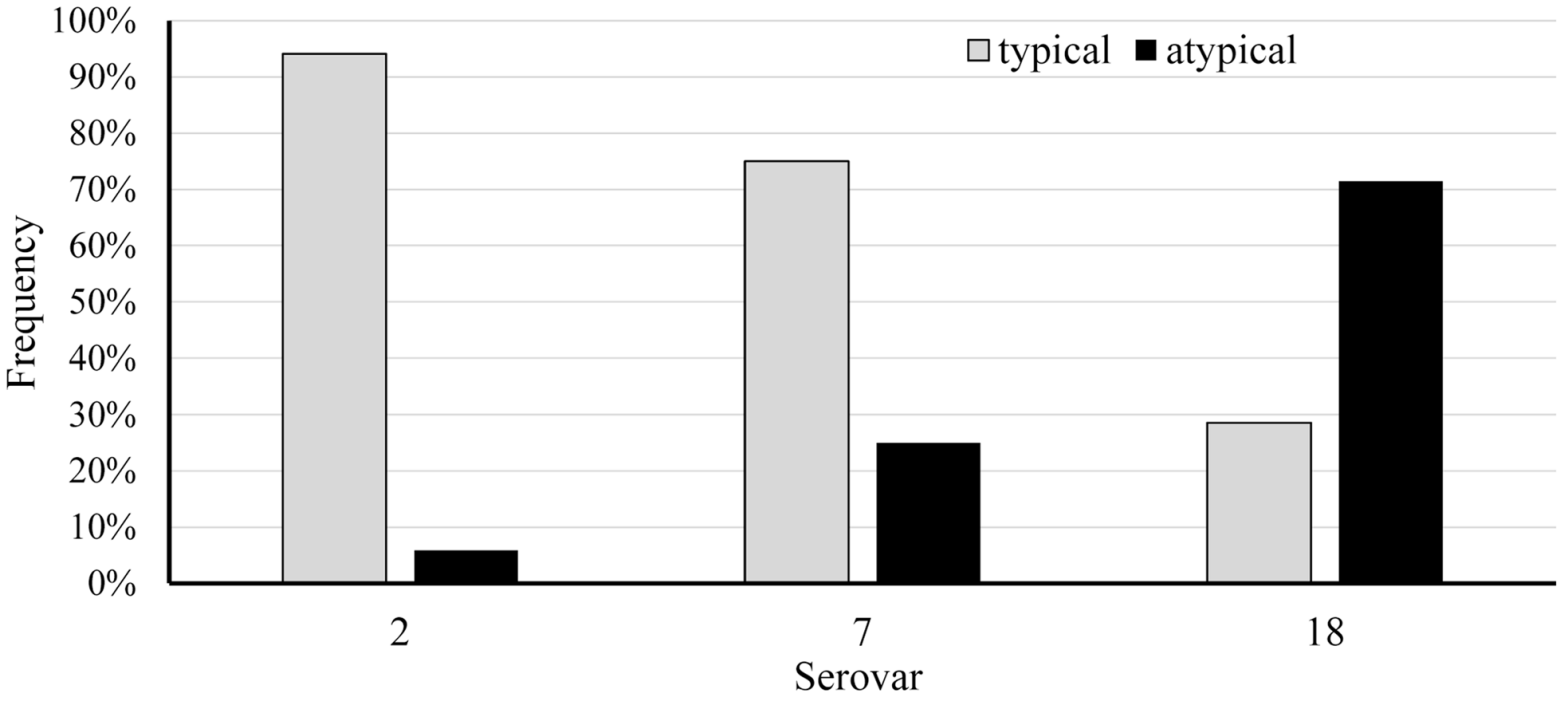
**Proportion of atypical** ***apx*** **profiles in relation to the serovar.**Serovar 2 (n = 136), Serovar 7 (n = 4), Serovar 18 (n = 7).

Notably, the typical serovar-specific *apx* gene profile found in most isolates was contrasted by a minority of 6.6% of isolates (n = 14) which showed different toxin profiles deviating from this general pattern. Eight of these fourteen isolates belonged to serovar 2, while five isolates were typed as serovar 18 and one isolate as serovar 7 (see Figure 2).

Compared to the representatives of serovar 18 classified as typical, all atypical isolates of this serovar (n = 5) showed non-hemolytic growth on sheep blood agar. Genotypically, only *apxIIICABD* was detected in all these isolates. In addition, one of these isolates showed NAD-independent growth. The additional strain collection included another representative of serovar 18 from 2009, which only carried *apxIIICABD* and showed non-hemolytic growth, too. The *apx*-examination of the eight atypical isolates of serovar 2 showed a heterogeneous phenotypic and genotypic picture. Four representatives of this group lacked the *apxIIICABD* operon, resulting in an *apx* profile typical for serovars 7, 12, 13 and the reference strains of serovars 17 and 18. These four isolates showed hemolysis and NAD-dependent growth on sheep blood agar. In another member of this group, the *apxICABD* operon and *apxIICA* were detected, which represents the toxin profile of serovars 1, 5, 9, 11 and 16. This isolate was NAD-dependent and hemolytic. In contrast, the remaining three isolates of the atypical serovar 2 group did not show any similarity to regular toxin profiles of other serovars. One of the strains showed *apxIBD* and the complete *apxIIICABD* operon, non-hemolytic and NAD-dependent growth. In the other two isolates, genes of all three *apx* operons (*apxIBD, apxIICA, apxIIICA*), NAD-dependence and hemolysis on sheep blood agar were detected. Finally, the atypical serovar 7 isolate had a complete *apxICABD* operon but no ApxII coding genes. This strain grew NAD-dependent and showed hemolysis on blood agar. Results for atypical isolates are summarized in Table 2.

**Table 2:** Pheno- and genotype of selected isolates with atypical *apx* gene profile, biovar II phenotype or of only recently described serovars in the isolate collections

Lab ID	Biovar	Serovar	*apxIBD*	*apxICA*	*apxIICA*	*apxIIIBD*	*apxIIICA*	*apx*-profile typical for Serovar	Hemolysis	*nadV*
4065/1/18	I	2	+	–	+	–	–	7, 12, 13, 17, 18	Yes	–
1072/1/19	I	2	+	–	+	–	–	7, 12, 13, 17, 18	Yes	–
4282/1/19	I	2	+	–	+	–	–	7, 12, 13, 17, 18	Yes	–
4630/2/11	I	2	+	–	+	–	–	7, 12, 13, 17, 18	Yes	–
261/1/19	I	2	+	–	+	–	+	Unknown	Yes	–
4980/15	I	2	+	–	+	–	+	Unknown	Yes	–
572/2/12	I	2	+	–	–	+	+	Unknown	No	–
3697/2/10	I	2	+	+	+	–	–	1, 5, 9, 11, 16	Yes	–
5074/4/14	I	7	+	+	–	–	–	10, 14	Yes	–
1567/1/10	I	16	+	+	+	–	–	1, 5, 9, 11, 16	Yes	–
730/1/12	I	16	+	+	+	–	–	1, 5, 9, 11, 16	Yes	–
3726/6/15	I	18	+	–	+	–	–	7, 12, 13, 17, 18	Yes	–
1565/10	I	18	+	–	+	–	–	7, 12, 13, 17, 18	Yes	–
5830/11	I	18	–	–	–	+	+	Unknown	No	–
5622/2/11	I	18	–	–	–	+	+	Unknown	No	–
3677/1/10	I	18	–	–	–	+	+	Unknown	No	–
95/14	I	18	–	–	–	+	+	Unknown	No	–
**2470/3/16**	**II**	**18**	**–**	**–**	**–**	**+**	**+**	**Unknown**	**No**	**+**
1739/1/18	I	n.t.	+	+	+	–	–	1, 5, 9, 11, 16	Yes	–
5057/1/18	I	n.t.	+	+	+	–	–	1, 5, 9, 11, 16	Yes	–
2717/19	I	n.t.	+	+	+	–	–	1, 5, 9, 11, 16	Yes	–
2894/2/19	I	n.t.	+	+	+	–	–	1, 5, 9, 11, 16	Yes	–
6961/1/10	I	n.t.	+	+	+	–	–	1, 5, 9, 11, 16	Yes	–
5419/1/10	I	n.t.	+	+	+	–	–	1, 5, 9, 11, 16	Yes	–
5536/3/17	I	n.t.	+	–	+	–	–	7, 12, 13, 17, 18	Yes	–
158/2/19	I	n.t.	+	–	+	–	–	7, 12, 13, 17, 18	Yes	–
1452/6/12	I	n.t.	+	–	+	–	–	7, 12, 13, 17, 18	Yes	–
3809/4/09*	I	18	–	–	–	+	+	Unknown	No	–
1542/01*	**II**	**2**	**+**	**–**	**+**	**–**	**–**	**7, 12, 13, 17, 18 / 2, 4, 6, 8, 15 (Biovar II)**	**Unknown**	**+**
1617/01*	**II**	**2**	**+**	**–**	**+**	**–**	**–**		**Unknown**	**+**

Biovar II isolates are shown in bold and isolates from the additional collection are marked with an asterisk. The latter therefore harbor a typical *apx* pattern.

### Biovar

In terms of biovar, only one *A.* *pleuropneumoniae* isolate was classified as biovar II by growing on BHI agar and independent of a nurse. The isolate was typed as serovar 18 and the assignment to biovar II could be confirmed by the detection of *nadV*. This isolate showed abundant growth on both chocolate and sheep blood agar (non-hemolytic). In this biovar II isolate only *apxIIICABD* was detected.

In the additional strain collection, the *nadV* gene was detected in two representatives of serovar 2, which had been stored as NAD-independent representatives of *A.* *pleuropneumoniae* at the Institute for Microbiology, and thus the classification as biovar II was molecularly confirmed. A phenotypic evaluation of these isolates could not be performed due to unsuccessful re-activation of the freeze-dried cultures. No *apxIIICABD* operon was detected in these two isolates. A detailed list of the geno- and phenotypic characteristics of specific isolates described above is given in Table 2.

### Systemic isolates

**Figure 3:**
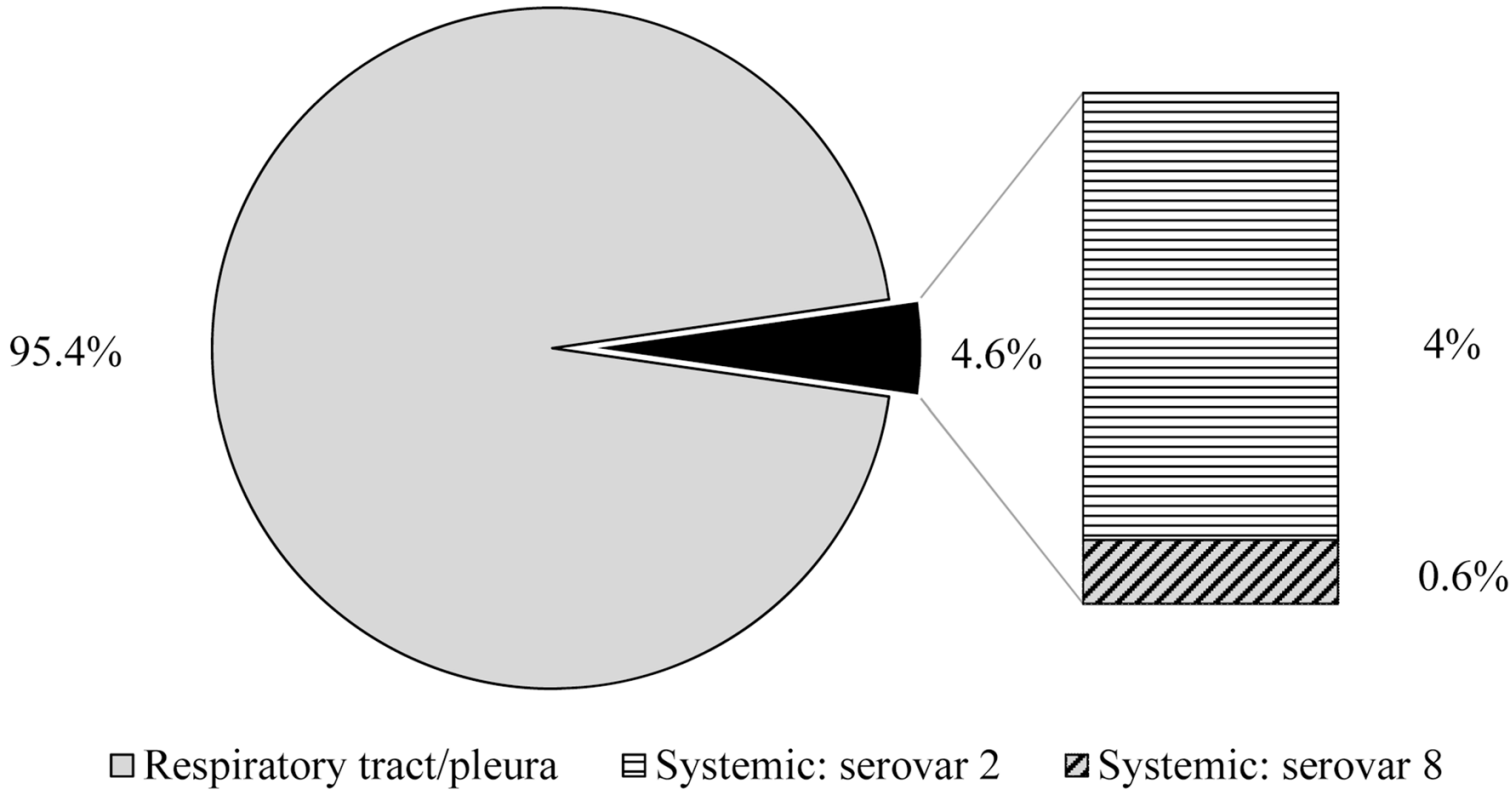
**Occurrence of serovars with regard to the site of isolation.** Out of all isolates with known organ of origin, eight isolates originated from systemic sites and were typed as serovar 2 (n = 7) and serovar 8 (n = 1).

Among all isolates of which the sampling site was known (n = 175), only 4.6% (n = 8) were not detected in the respiratory tract but in systemic localizations, such as joints (n = 6) or the central nervous system (n = 2, see Figure 3). In addition, one isolate from the additional collection was isolated from a joint of a diseased animal. Seven of the eight isolates from systemic localizations belonged to serovar 2 (matching with the overall serovar distribution of the collection) and, thus, represented 6% of all serovar 2 isolates with known organ of origin (n = 115). The systemic isolate of the additional collection from a joint was also typed as serovar 2. The remaining representative from systemic localizations belonged to serovar 8.

All systemic isolates showed typical compositions of Apx toxin genes. Furthermore, no correlation of a systemic manifestation with the age of the animal was detected, as both young animals and animals in the fattening period were affected.

## Discussion

### Serotyping

Serotyping is the most important tool to differentiate isolates of *A.* *pleuropneumoniae*. Up to now, 18 serovars are known, which can be differentiated molecularly with the method according to Bossé et al. [11]. For this, the serovars 9 and 11 are an exception, as they only differ by a single nucleotide in the serovar-specific genes *cpsEF*. Isolates of these serovars are therefore typed as serovar 9/11 [11].

With regard to the serovar frequency, only few studies exist at all, which are partially outdated. Serovar 2 was described as the dominating serovar in Hungary, Belgium and, among others, in Denmark and the Netherlands [23–25], while the important role of serovar 8 in Great Britain and serovar 7 in Spain was demonstrated [26, 27]. In Australia, serovar 15 is most commonly detected whereas in Canada serovars 5 & 7 and in South Korea serovars 1 & 5 are most frequent [28–30]. Our results support a dominance of serovar 2 in central European regions and highlight geographical differences within Europe as compared to the Iberian Peninsula or Great Britain as well as across other continents. This highlights the need for serotyping in order to select appropriate vaccines for porcine pleuropneumonia in different countries or regions as long as no efficient serotype-independent vaccine strategies are available. Publications on the serovar distribution in Germany were outdated and indicated a geographical difference between the two German states at that time, highlighting the predominant role of serovars 3 and 7 in the Federal Republic of Germany and of serovar 2 in the German Democratic Republic [31, 32]. Our isolate collection represents the density of pigs in farms across Germany quite well though it was not obtained from a randomized sample volume which, however, excludes statistical analysis (see Additional file 1). Still, based on this collection, a reduction of serovar diversity with the strong dominance of only one serovar, namely serovar 2, was observed. This development in favor of this serovar is also evident temporally within the decade (2010-2019) covered by our study. It has to be noticed that the identification of a shift is based on the chosen grouping of our isolates into the three groups containing one-third of isolates each and is most evident when comparing cohort A (oldest isolates) and cohort C (latest isolates). In the context of changes in the serovar frequency, a shift in the serovar prevalence of *A.* *pleuropneumoniae* has been described in Canada, where the majority of isolates from 2011 to 2014 were either serovar 5 or 7, compared to the predominance of serovar 1 in isolates detected between 1970 and 1990 [28, 33]. A comparable shift of the dominant serovars has also been described in other bacteria such as *Streptococcus* *suis* and *Streptococcus pneumoniae*, which some authors explain by the application of serovar-specific vaccination measures resulting in the displacement of the serovars used in the vaccines [34, 35]. However, for *A. pleuropneumoniae* the increasing dominance of serovar 2 is interesting, since three of four commercially available, licensed vaccines on the German market contain capsule antigens of serovar 2. Also autogenous vaccines many times would be based on a serovar 2 isolate. In the context of the assumption of Klinkenberg et al., that disease outbreaks are more likely to be triggered by external factors in already subclinically infected animals rather than by new infections [36], the increased detection of moderately virulent representatives of *A.* *pleuropneumoniae* in clinical outbreaks would be explainable. As a consequence of the “external trigger-theory” proposed by Klinkenberg et al., strains belonging to serovars 2, 4, 6, 8 and 15 of biovar I may either have a bias for the carrier status since these serovars do not secrete Apx I or the host may be more adapted to these subtypes of the bacterium. However, this assumption does not explain the increased detection of serovar 2 among other moderately virulent serovars in our collection. As a general reduction of agricultural farms and breeding lines is seen and infrastructural improvements in the past years facilitates animal transport over longer distances, the spreading of single strains and serovars may be explainable. This is supported by the observation that serovar 2 plays an important role in central European countries, while in other regions that are geographically peripheral like Spain or UK other serovars are more prevalent. As our data are not suitable to clarify this issue, more studies are needed in this context.

When looking at the serovar distribution in our collection, the detection of the two only recently described serovars 16 and 18 illustrates the importance of molecular serotyping including primers for serovar 16, 17 and 18. Otherwise corresponding isolates would be classified as non-typable. The representatives of serovar 16 were isolated in 2010 and 2012, those of serovar 18 in 2009–2011 and 2014–2016. These findings correspond with the original description of the “new” serovars in strains isolated as early as 1996 in Denmark (serovar 18) and 2012–2014 in Hungary (serovar 16) [9, 10]. Since all isolates characterized in this study were confirmed as *A. pleuropneumoniae* by detection of *apxIVA*, the few non-typable isolates may contain mutated primer binding sites in the capsule locus impeding molecular serotyping. The occurrence of no significant similarity sequences (NSSS) was reported in *Glaesserella* *parasuis* and is likely to occur in other bacteria like *A. pleuropneumoniae*, too [37]. Therefore, NSSS may entail the risk of a false non-typability with molecular methods. On the other hand, non-typability using molecular methods may also indicate the occurrence of genuinely new serovars which have yet to be identified.

### Apx toxin gene profiles

As a consequence of the repeatedly reported detection of atypical isolates in *A.* *pleuropneumoniae* [17–21], an additional aim of this study was the characterization of the *apx*-profiles in order to check the validity of the alleged serovar-specific association of these toxin genes. Among others, strains with atypical toxin gene profiles were found in Switzerland, Korea, Japan and Australia, belonging to a wide range of different serovars [17–21]. The toxin typing performed here revealed that only 7% of the examined isolates deviated from the expected *apx*-profile. This was particularly noticeable in isolates of serovar 18, as the majority of this serovar in our collection had toxin profiles differing from the *apx* profile of the reference strain (*apxIIICABD*-operon instead of *apxIBD* & *apxIICA*). Thus, the relative proportion of atypical isolates in relation to the serovar is highest in this serovar with 75% (n = 6/8). So far, only eight isolates of this serovar in addition to the reference strain are published, which all harbour *apxIBD* and *apxIICA* [10]. The representativity of *apx* characteristics of the serovar 18 reference strain (strain 7311555) could thus be questioned. In order to clarify this situation, more isolates of this serovar are needed for *apx* characterization.

The high proportion of atypical serovar 2 isolates with respect to *apx* toxin gene profiles is not surprising regarding the generally high frequency of this serovar. Some of the unusual serovar 2 *apx* toxin gene profiles have been described before (for an overview see [7]). Although it is known that biovar II strains of *A. pleuropneumoniae* usually lack the *apxIII* operon this was also seen in four of our biovar I serovar 2 isolates as described previously [12, 15]. Three more *apx* patterns were seen in our collection and their overall variety suggests that isolates are epidemiologically unlinked and none represents a spreading genotype with a selective advantage.

### Biovar

Most of the isolates examined in this study belonged to biovar I. The NAD-dependent growth was even observed in the only isolate of serovar 13, a serovar generally considered to be NAD-independent. However, this was described in North America before [7, 38]. Moreover, it can be confidentially excluded that biovar II isolates would have been misidentified as *A. suis* since in our laboratory such isolates would undergo biochemical identification including distinguishing reactions like fermentation of salicin acid, arabinose acid, trehalose acid, cellobiose acid, and melibiose acid as well as aesulin hydrolysis. NAD-independent isolates with a biochemical profile typical for *A. pleuropneumoniae* would then be confirmed to the species by *apxIV*-PCR. Biovar II isolates of *A.* *pleuropneumoniae* are, in contrast to biovar I, able to synthesize NAD from Nam (nicotinamide) using the enzymes NadV and NadR. Thus, NAD independence was shown to be due to the presence of the gene *nadV* [39, 40]. In most cases, field isolates of *A.* *pleuropneumoniae* belong to biovar I, although regional differences were observed with only occasionally involvement of biovar II isolates in disease events [13, 15, 27]. An exception is the increased detection in clinically affected pigs in Spain with a quarter of the isolates belonging to this phenotype. More than two-thirds of those isolates belonged to serovar 7, a serovar less frequently detected elsewhere in Europe. The perceived lower virulence of biovar II isolates has been explained by the universal lack of ApxIII formation compared to isolates of the same serovar and biovar I [15, 16, 41]. The lack of the *apxIIICABD* operon was evident in the two *nadV*-positive serovar 2 biovar II isolates. However, in the serovar 18 biovar II isolate (*nadV*-positive) just and only the *apxIIICABD* operon was detected. All other serovar 18 isolates that harbored this *apx* profile were classified as biovar I. To the best of our knowledge, this is the first evidence of a biovar II isolate harboring the *apxIIICABD* operon.

### Systemic isolates

Systemic spreading and the ability of *A.* *pleuropneumoniae* to cause disease besides classical pleuropneumonia is not well understood, but documented in the literature [3–5]. In our investigation, nine isolates originated from systemic localizations. All systemic isolates were characterized as serovar 2 or serovar 8 showing the expected *apx* pattern. They therefore belong to the moderately virulent subtypes of this bacterial species. The systemic isolates identified in other studies also belong to low to moderate virulent serovars (2, 7 or 8), too [3–5]. This prompts the notion that representatives with reduced virulence may be more prone to be found in systemic sites, which may be due to a more moderate course of disease with enough time to develop lesions also in other organs. On the other hand, highly virulent isolates are more likely to cause acute illness and death and animals may succumb to disease before bacteremia results in macroscopic lesions in other organs. In those cases, presence of the bacteria in other organs would go undetected by necropsy and bacterial culture. More research is needed to clarify time courses and routes of spreading in relation to the infecting serovar.

The molecular methods of sero- and *apx*-typing used in this study were shown to be good tools for characterizing most field isolates of *A.* *pleuropneumoniae*. In view of the results shown here, the predominant role of biovar I serovar 2 strains and the almost constitutive endowment of this serovar with Apx II and Apx III suggests that a convergent evolution in favor of this specific, moderate virulent subtype is ongoing in central Europe. Moderate virulence could be regarded as a survival strategy rendering the spreading in pig populations comparatively easy since transmission is negatively affected by severity of disease [42]. This seems to be advantageous, as *A.* *pleuropneumoniae* has a low environmental tenacity and is dependent on life in its host animal.

## Supplementary information

**Additional file 1. Geographic origin of isolates (A), analysed with the software package “Das Postleitzahlen-Diagramm 4.0” by Klaus Wessiepe (**www.Klaus-Wessiepe.de**) licensed for "Institut für Mikrobiologie, Tierärztliche Hochschule Hannover“, 2007) and pig population in Germany (B; map from Fleischatlas, 2016, by Heinrich-Böll-Stiftung, CC BY-SA 3.0 DE).**

**Additional file 2. Raw data for 213 German** ***A.*** ***pleuropneumoniae*** **isolates (2010–2019) and 5 additional isolates (1999–2009).**

## Data Availability

The raw data set is included as Additional file 2.

## Abbreviations

*A*.: *Actinobacillus*
*apx*: *Actinobacillus pleuropneumoniae* RTX
BHI agar: Brain heart infusion agar
NAD: Nicotinamide adenine dinucleotide
PCR: Polymerase chain reaction
PRDC: Porcine respiratory disease complex
RTX: Repeats in toxin
µl: Microliter
µM: Micromole
