# Supplementary figures and images for: Sero- and apx-typing of German Actinobacillus pleuropneumoniae field isolates from 2010 to 2019 reveals a predominance of serovar 2 with regular apx-profile
Source: Vet Res. 2021 Jan 20;52:10. doi: 10.1186/s13567-020-00890-x (PMC7818768; doi:10.1186/s13567-020-00890-x)

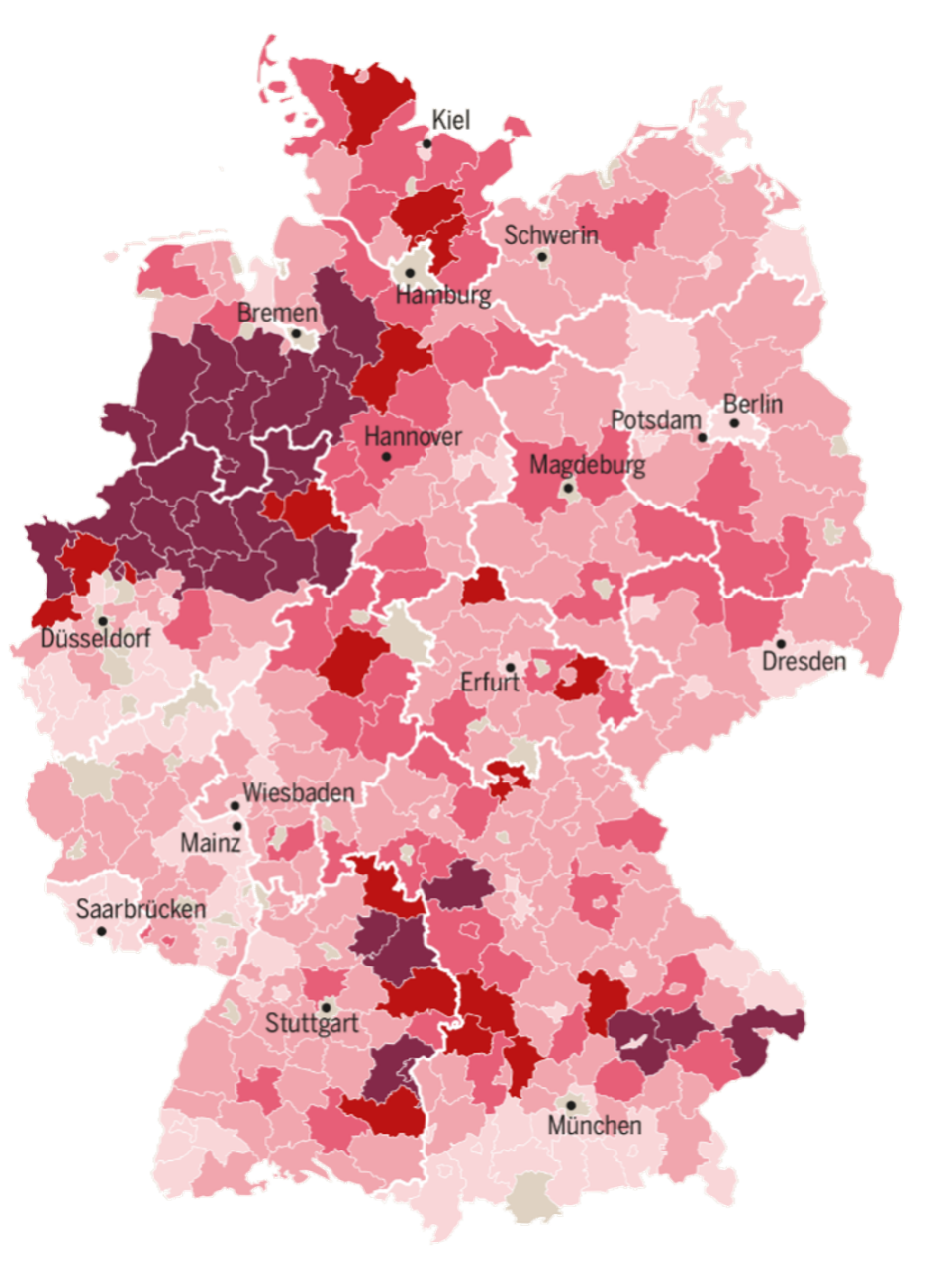

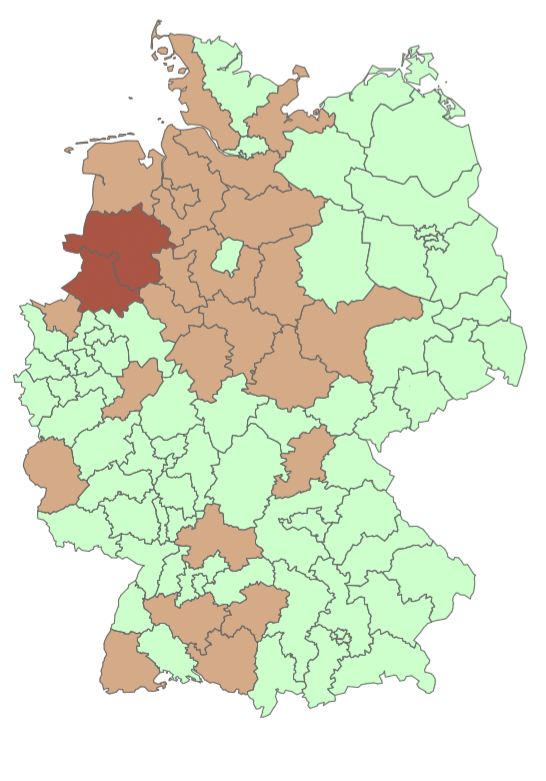


**origin and number of isolates used in this study**

**pig density per 100 hectar in Germany**


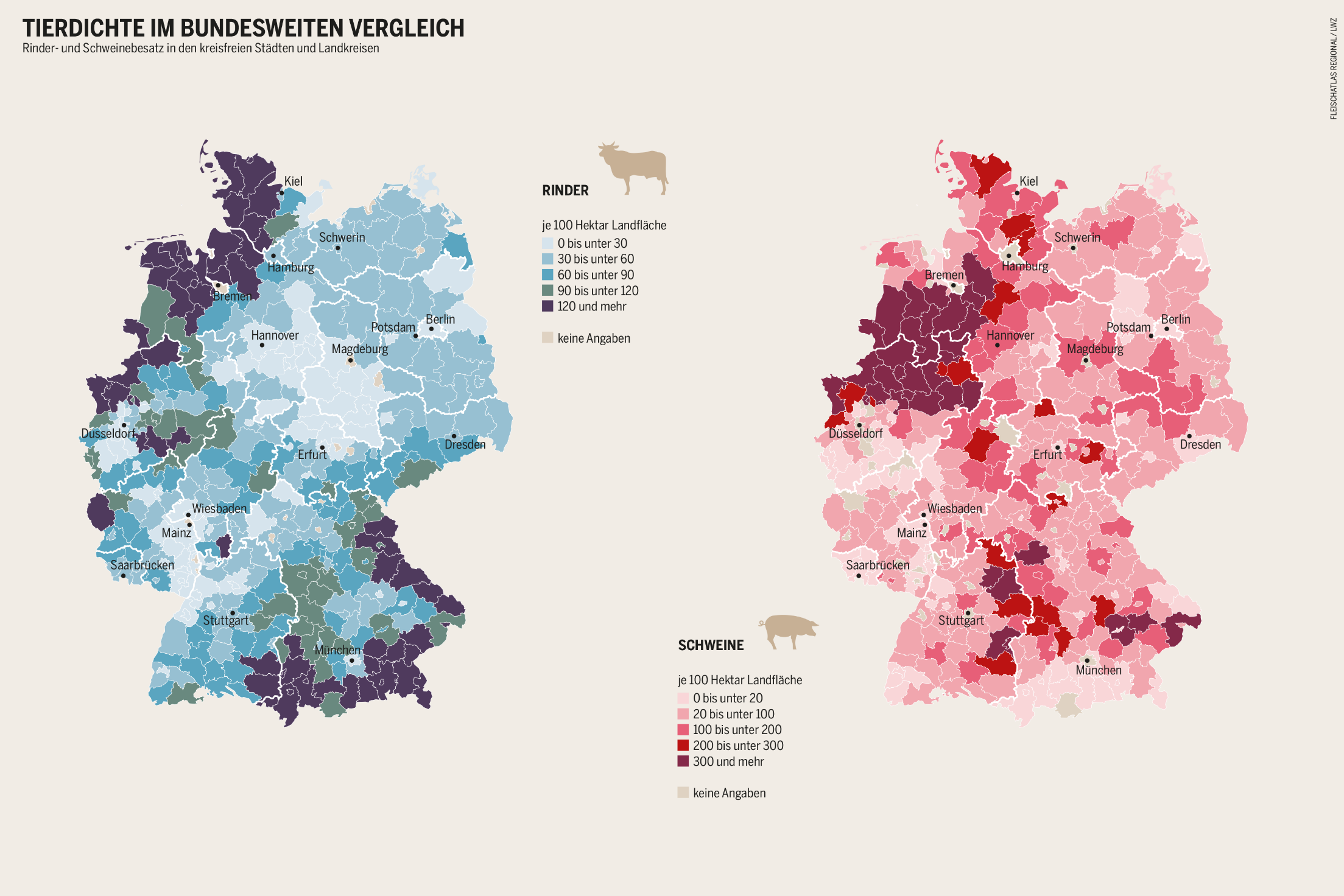


0-20

21 -100

101-200

201-300

>300

unknown

A

B

Supplement: Supplementary file 1 — Additional file 1. Geographic origin of isolates (A), analysed with the software package “Das Postleitzahlen-Diagramm 4.0” by Klaus Wessiepe (www.Klaus-Wessiepe.de) licensed for "Institut für Mikrobiologie, Tierärztliche Hochschule Hannover“, 2007) and pig population in Germany (B; map from Fleischatlas, 2016, by Heinrich-Böll-Stiftung, CC BY-SA 3.0 DE). [file 13567_2020_890_MOESM1_ESM.docx]
